# Supplementary figures and images for: How do host population dynamics impact Lyme disease risk dynamics in theoretical models?
Source: PLoS One. 2024 May 9;19(5):e0302874. doi: 10.1371/journal.pone.0302874 (PMC11081252; doi:10.1371/journal.pone.0302874)

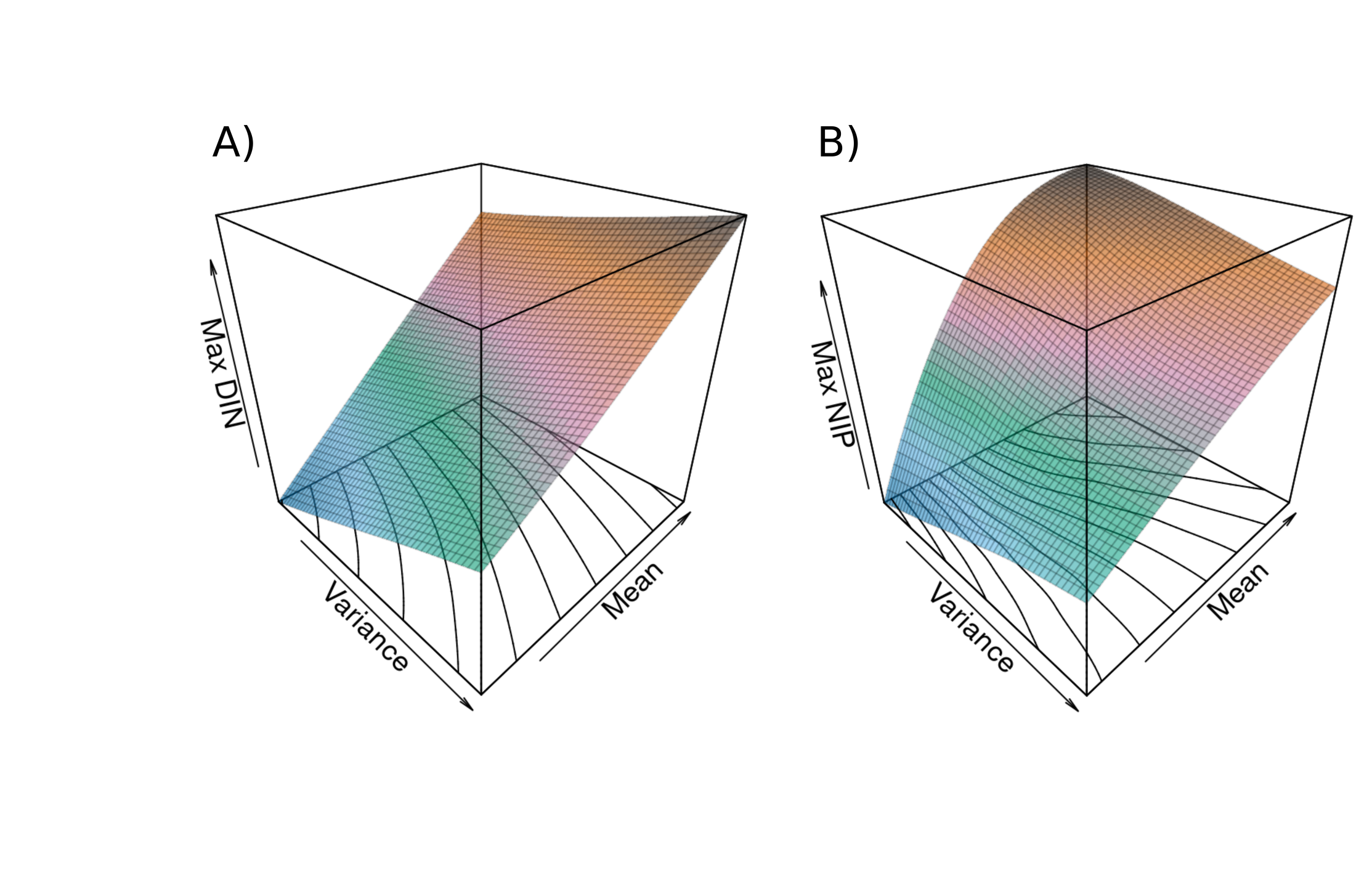

Supplement: S1 Fig — A) shows the response of maximum DIN to mouse variation and mean density. The relationship to mean mouse density is positive and linear, as is the relationship to mouse variance. Contour lines plotted on the variance × mean plane and coloring reveal surface features. B) shows the response of maximum NIP to mouse variation and mean density. There is a nonlinear positive relationship between maximum NIP and mean mouse density. The relationship between mouse population variance and maximum NIP is inconsistent, with higher variance increasing maximum NIP at the low mean mouse density. At higher mean densities, maximum NIP transitions to decreasing with variance. Contour lines and coloration again reveal surface features. (TIF) [file pone.0302874.s001.tif]

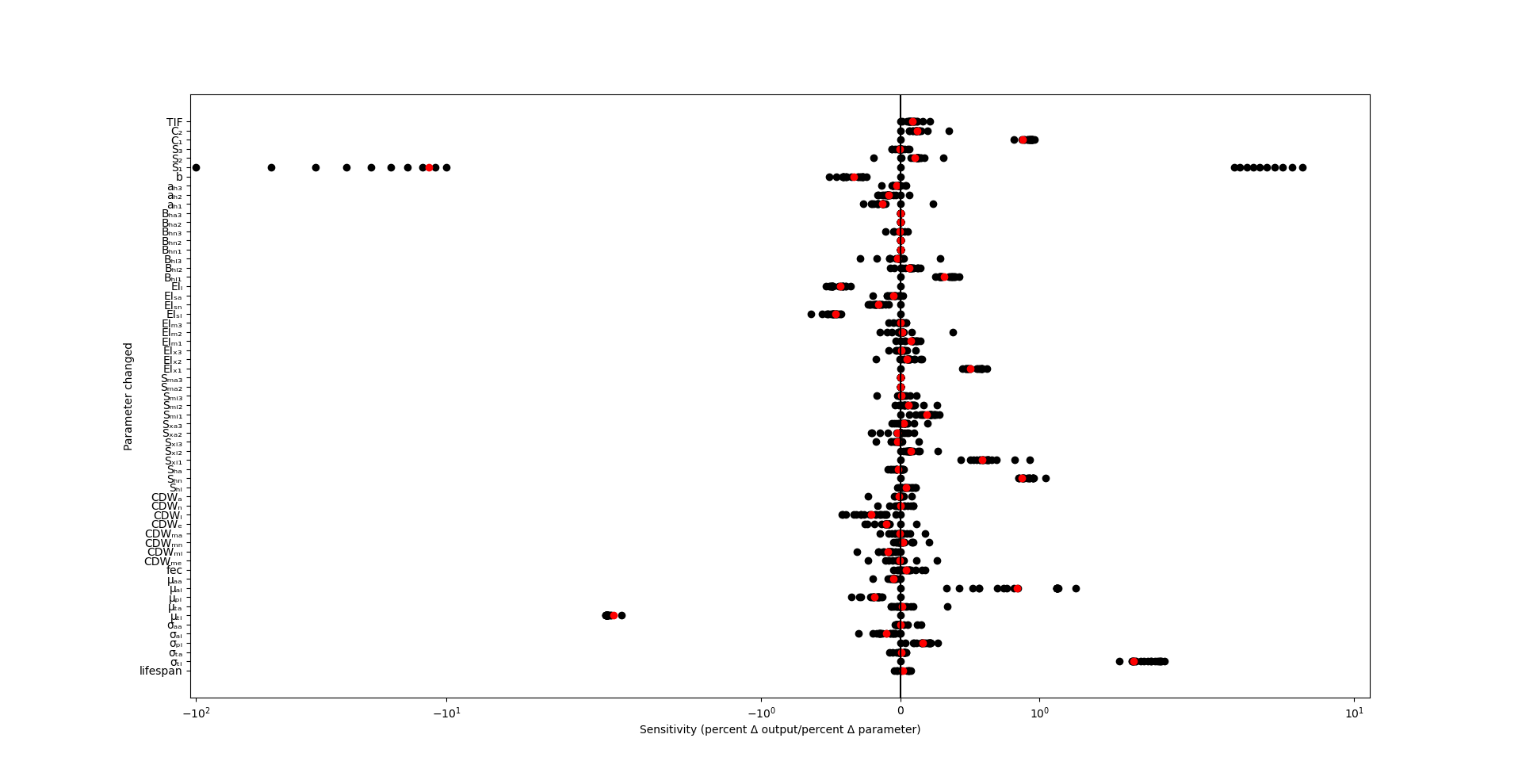

Supplement: S2 Fig — This figure depicts the results of the sensitivity analysis we conducted. Parameters changed appear on the y-axis, with each point on the x-axis indicating the sensitivity measures (percent change in output/percent change in parameter). Red dots indicate the mean sensitivity of each parameter. (TIF) [file pone.0302874.s002.tif]
