# Supplementary material for: How do host population dynamics impact Lyme disease risk dynamics in theoretical models?
Source: PLoS One. 2024 May 9;19(5):e0302874. doi: 10.1371/journal.pone.0302874 (PMC11081252; doi:10.1371/journal.pone.0302874)
Supplement: S1 Table — This table presents summary statistics for disease risk metrics, grouped by different levels of mouse population variance. (PDF) [file pone.0302874.s003.pdf]

Disease Metric	variance	min	Q1	mean	median	Q3	max
mean DIN	0	570	2031	3351	3394	4694	5968
	1-33	579	1927	3285	3270	4588	7055
	34-66	663	2099	3280	3132	4327	8047
	67-99	694	2412	3518	3327	4495	8958
min DIN	0	34	125	207	210	291	367
	1-33	34	121	205	204	286	437
	34-66	37	131	205	197	273	505
	67-99	39	147	217	204	281	639
max DIN	0	1829	6447	10677	10805	14981	18980
	1-33	1869	7120	11304	11400	15442	24159
	34-66	2438	9252	13122	12995	16813	28904
	67-99	2715	11362	15319	15109	19070	33706
amp DIN	0	1796	6322	10470	10595	14690	18613
	1-33	1835	7007	11099	11193	15145	23760
	34-66	2397	9114	12917	12804	16557	28412
	67-99	2676	11185	15102	14896	18804	33067
mean DON	0	1790	4101	6075	6173	8109	9997
	1-33	1809	4110	6143	6168	8099	11793
	34-66	2040	4717	6466	6308	8092	13695
	67-99	2115	5441	7113	6882	8693	15296
min DON	0	185	356	506	513	660	800
	1-33	186	361	513	514	662	956
	34-66	195	397	534	525	661	1094
	67-99	200	441	575	556	699	1384
max DON	0	9625	18629	26569	26923	34696	42160
	1-33	9711	21187	28779	28994	36479	55245
	34-66	11298	27541	34850	34835	41885	69202
	67-99	12451	33688	41572	41497	49346	76826
amp DON	0	5618	12297	18258	18495	24348	29964
	1-33	5680	13962	19648	19768	25348	38853
	34-66	6746	18062	23510	23463	28733	46425
	67-99	7450	22130	27853	27758	33435	54137
mean NIP	0	0.32	0.49	0.53	0.55	0.58	0.6
	1-33	0.32	0.46	0.51	0.53	0.57	0.6
	34-66	0.32	0.43	0.48	0.48	0.53	0.61
	67-99	0.33	0.42	0.46	0.46	0.5	0.6
min NIP	0	0.3	0.36	0.38	0.38	0.4	0.41
	1-33	0.27	0.32	0.35	0.36	0.38	0.41
	34-66	0.24	0.29	0.32	0.32	0.34	0.41
	67-99	0.22	0.28	0.3	0.3	0.32	0.4
max NIP	0	0.32	0.53	0.57	0.6	0.63	0.65
	1-33	0.32	0.52	0.56	0.59	0.62	0.66
	34-66	0.34	0.5	0.55	0.56	0.6	0.66
	67-99	0.35	0.5	0.54	0.54	0.59	0.66
amp NIP	0	0.03	0.17	0.19	0.21	0.23	0.24
	1-33	0.03	0.19	0.21	0.23	0.24	0.28
	34-66	0.05	0.21	0.23	0.24	0.26	0.31
	67-99	0.07	0.21	0.24	0.24	0.27	0.33
