# Supplementary material for: How do host population dynamics impact Lyme disease risk dynamics in theoretical models?
Source: PLoS One. 2024 May 9;19(5):e0302874. doi: 10.1371/journal.pone.0302874 (PMC11081252; doi:10.1371/journal.pone.0302874)
Supplement: S2 Table — Tick life and developmental stages represented in the model. (PDF) [file pone.0302874.s004.pdf]

stage class	description	duration
Eggs	Beginning (1st stage) of tick development	52 weeks
Hardening larvae	Newly hatched larval (2nd stage) ticks, which must harden	1 week
Questing larvae	Larvae which have not fed, and are able to quest for hosts	52 weeks
On-host larvae	Larvae which have found, and are currently feeding from, a host	52 weeks
Engorged larvae	Larvae which have fed and now must develop into nymphs	52 weeks
Hardening nymphs	Newly hatched nymphal (3nd stage) ticks, which must harden	1 week
Questing nymphs	Nymphs which have not fed, and are able to quest for hosts	52 weeks
On-host nymphs	Nymphs which have found, and are currently feeding from, a host	52 weeks
Engorged nymphs	Nymphs which have fed and now must develop into nymphs	52 weeks
Hardening adults	Newly hatched adult (4th stage) ticks, which must harden	1 week
Questing adults	Adults which have not fed, and are able to quest for hosts	52 weeks
On-host adults	Adults which have found, and are currently feeding from, a host	52 weeks
Engorged adults	Adults which have fed and now must develop into nymphs	52 weeks
