# Supplementary material for: How do host population dynamics impact Lyme disease risk dynamics in theoretical models?
Source: PLoS One. 2024 May 9;19(5):e0302874. doi: 10.1371/journal.pone.0302874 (PMC11081252; doi:10.1371/journal.pone.0302874)
Supplement: S3 Table — Tick demographic processes represented in the model. (PDF) [file pone.0302874.s005.pdf]

stage category	processes
Egg	survival, development
Free living unfed	survival, questing
Free living fed	survival, development
Hardening	survival
On-host	survival, infection, transmission
