# Supplementary material for: How do host population dynamics impact Lyme disease risk dynamics in theoretical models?
Source: PLoS One. 2024 May 9;19(5):e0302874. doi: 10.1371/journal.pone.0302874 (PMC11081252; doi:10.1371/journal.pone.0302874)
Supplement: S5 Table — (PDF) [file pone.0302874.s007.pdf]

Parameter name	Description	Value	Citation
$S_{x,i}$	Maximum on-host immature survival for each host (mice, medium, deer)	0.6	[1]
$S_{x,a}$	Maximum on-host adult survival for each host (mice, medium, deer)	(0, 0.6, 0.7746)	[1]
$S_{m,i}$	Minimum on-host immature survival for each host (mice, medium, deer)	0.2	[1]
$S_{m,a}$	Minimum on-host adult survival for each host (mice, medium, deer)	(0, 0.2, 0.4474)	[1]
EI_{max}	Maximum engorgement index for each host (mice, medium, deer), and all on host stages	(0.75, 4, 300)	[1]
EI_{min}	Minimum engorgement index for each host (mice, medium, deer), and all on host stages	(0.15, 0.8, 60)	[1]
EI_{scl}	Engorgement index allometric scaling coefficient for (larval, nymphal, adult) tick density	(0.0021, 0.014, 1)	[1]
EI_{loss}	Engorgement index weekly loss	0.44	[1]
$B_{h,l}$	Host larval burdens for each host (mice, medium, deer)	(100, 200, 1000)	[1]
$B_{h,n}$	Host nymphal burdens for each host (mice, medium, deer)	(20, 100, 500)	[1]
$B_{h,a}$	Host adult burdens for each host (mice, medium, deer)	(0, 20, 100)	[1]
a_h	Basal host finding rate for each host (mice, medium, deer), and all tick stages	(0.01, 0.025, 0.05)	[1]
b	Host finding rate scaling for all tick stages and species	0.515	[1]
S_{mice}	Mouse survival	0.9808	*
S_{med}	Medium host survival	0.9904	*
S_{deer}	Deer survival	0.9936	*
C_{mice}	Mouse competency	0.75	[2, 3]
C_{med}	Medium host competency	0.5	[3–5]
C_{deer}	Deer competency	0	[4, 6, 7]
TIF	Tick infectivity factor, tick-to-host transmission	0.9	[1]

* values chosen as part of study.

** Initial conditions taken from week 0 of the last year of a simulation with mean mouse density set to 40 and no variation.
