# Supplementary material for: How do host population dynamics impact Lyme disease risk dynamics in theoretical models?
Source: PLoS One. 2024 May 9;19(5):e0302874. doi: 10.1371/journal.pone.0302874 (PMC11081252; doi:10.1371/journal.pone.0302874)
Supplement: S1 File — The equations representing tick, host, and infection processes in the model are presented here. See S4 and S5 Tables for parameter names and purposes. (PDF) [file pone.0302874.s008.pdf]

1. $$E_{wk+1} = \begin{cases} 0 \text{ if } CDW < CDW_{dev,a} \\ fec \cdot (EA_{wk} + IEA_{wk}) \text{ if } CDW >= CDW_{dev,a} \end{cases} + \left(E_{wk} - \begin{cases} 0 \text{ if } CDW < CDW_{dev,e} \\ E_{wk} \text{ if } CDW >= CDW_{dev,e} \end{cases} \right) \cdot N(\mu_{t,i}, \sigma_{t,i}) \cdot N(\mu_{p,i}, \sigma_{p,i})$$

2. $$HL_{wk+1} = \begin{cases} 0 \text{ if } CDW < CDW_{dev,e} \\ E_{wk} \text{ if } CDW >= CDW_{dev,e} \end{cases} \cdot S_{h,l}$$

3. $$QL_{wk+1} = (QL_{wk} - \textstyle\sum_{x=1}^{3}(QL_{wk} \cdot (a_x \cdot H_x)^b) \cdot N(\mu_{a,i}, \sigma_{a,i})) \cdot (N(\mu_{t,i}, \sigma_{t,i}) \cdot N(\mu_{p,i}, \sigma_{p,i}))$$

4. $$OL_{wk+1} = \textstyle\sum_{x=1}^{3}(QL_{wk} \cdot (a_x \cdot H_x)^b) \cdot N(\mu_{a,i}, \sigma_{a,i}) \cdot (N(\mu_{t,i}, \sigma_{t,i}) \cdot N(\mu_{p,i}, \sigma_{p,i}))$$

5a. $$EL_{wk+1} = \left(EL_{wk} - \begin{cases} 0 \text{ if } CDW < CDW_{dev,l} \\ EL_{wk} \text{ if } CDW >= CDW_{dev,l} \end{cases} \right) \cdot N(\mu_{t,i}, \sigma_{t,i}) \cdot N(\mu_{p,i}, \sigma_{p,i}) + \textstyle\sum_{x=1}^{3} \left(OL_{wk} \cdot \begin{cases} S_{x,i} \text{ if } EI_{l,wk} > EI_{max} \\ \frac{S_{m,i} - S_{x,i}}{EI_{max} - EI_{min}} \text{ if } EI_{min} < EI_{l,wk} < EI_{max} \\ S_{m,i} \text{ if } EI_{l,wk} < EI_{min} \end{cases} \cdot C_x (1 - \frac{IH_x}{IH_x + H_x}) \right)$$

5b. $$IEL_{wk+1} = \left(IEL_{wk} - \begin{cases} 0 \text{ if } CDW < CDW_{dev,l} \\ IEL_{wk} \text{ if } CDW >= CDW_{dev,l} \end{cases} \right) \cdot N(\mu_{t,i}, \sigma_{t,i}) \cdot N(\mu_{p,i}, \sigma_{p,i}) + \textstyle\sum_{x=1}^{3} \left(OL_{wk} \cdot \begin{cases} S_{x,i} \text{ if } EI_{l,wk} > EI_{max} \\ \frac{S_{m,i} - S_{x,i}}{EI_{max} - EI_{min}} \text{ if } EI_{min} < EI_{l,wk} < EI_{max} \\ S_{m,i} \text{ if } EI_{l,wk} < EI_{min} \end{cases} \cdot C_x \frac{IH_x}{IH_x + H_x} \right)$$

7a. $$HN_{wk+1} = \begin{cases} 0 \text{ if } CDW < CDW_{dev,n} \\ EL_{wk} \text{ if } CDW >= CDW_{dev,n} \end{cases} \cdot S_{h,n}$$

7b. $$IHN_{wk+1} = \begin{cases} 0 \text{ if } CDW < CDW_{dev,n} \\ IEL_{wk} \text{ if } CDW >= CDW_{dev,n} \end{cases} \cdot S_{h,n}$$

8a. $$QN_{wk+1} = (QN_{wk} - \textstyle\sum_{x=1}^{3}(QN_{wk} \cdot (a_x \cdot H_x)^b) \cdot N(\mu_{a,i}, \sigma_{a,i})) \cdot (N(\mu_{t,i}, \sigma_{t,i}) \cdot N(\mu_{p,i}, \sigma_{p,i}))$$

8b. $$IQN_{wk+1} = (IQN_{wk} - \textstyle\sum_{x=1}^{3}(IQN_{wk} \cdot (a_x \cdot H_x)^b) \cdot N(\mu_{a,i}, \sigma_{a,i})) \cdot (N(\mu_{t,i}, \sigma_{t,i}) \cdot N(\mu_{p,i}, \sigma_{p,i}))$$

9a. $$ON_{wk+1} = \textstyle\sum_{x=1}^{3}(QN_{wk} \cdot (a_x \cdot H_x)^b) \cdot N(\mu_{a,i}, \sigma_{a,i}) \cdot (N(\mu_{t,i}, \sigma_{t,i}) \cdot N(\mu_{p,i}, \sigma_{p,i}))$$

9b. $$ION_{wk+1} = \textstyle\sum_{x=1}^{3}(IQN_{wk} \cdot (a_x \cdot H_x)^b) \cdot N(\mu_{a,i}, \sigma_{a,i}) \cdot (N(\mu_{t,i}, \sigma_{t,i}) \cdot N(\mu_{p,i}, \sigma_{p,i}))$$

10a. $EN_{wk+1} =$

$$\left(EN_{wk} - \begin{cases} 0 \text{ if } CDW < CDW_{dev,n} \\ EN_{wk} \text{ if } CDW >= CDW_{dev,n} \end{cases}\right) \cdot N(\mu_{t,i}, \sigma_{t,i}) \cdot N(\mu_{p,i}, \sigma_{p,i}) +$$

$$\sum_{x=1}^{3} \left(ON_{wk} \cdot \begin{cases} S_{x,i} \text{ if } EL_{n,wk} > EI_{max} \\ \frac{S_{m,i}-S_{x,i}}{EI_{max}-EI_{min}} \text{ if } EI_{min} < EL_{n,wk} < EI_{max} \\ S_{m,i} \text{ if } EL_{n,wk} < EI_{min} \end{cases} \cdot C_x(1 - \frac{IH_x}{IH_x+H_x}) \right)$$

10a. $IEN_{wk+1} = \left(IEN_{wk} - \begin{cases} 0 \text{ if } CDW < CDW_{dev,n} \\ IEN_{wk} \text{ if } CDW >= CDW_{dev,n} \end{cases}\right) \cdot N(\mu_{t,i}, \sigma_{t,i}) \cdot$

$$N(\mu_{p,i}, \sigma_{p,i}) + \sum_{x=1}^{3} \left(ION_{wk} \cdot \begin{cases} S_{x,i} \text{ if } EL_{n,wk} > EI_{max} \\ \frac{S_{m,i}-S_{x,i}}{EI_{max}-EI_{min}} \text{ if } EI_{min} < EL_{n,wk} < EI_{max} \\ S_{m,i} \text{ if } EL_{n,wk} < EI_{min} \end{cases} \right) +$$

$$\sum_{x=1}^{3} \left(ON_{wk} \cdot \begin{cases} S_{x,i} \text{ if } EL_{n,wk} > EI_{max} \\ \frac{S_{m,i}-S_{x,i}}{EI_{max}-EI_{min}} \text{ if } EI_{min} < EL_{n,wk} < EI_{max} \\ S_{m,i} \text{ if } EL_{n,wk} < EI_{min} \end{cases} \cdot C_x(\frac{IH_x}{IH_x+H_x}) \right)$$

11a. $HA_{wk+1} = \begin{cases} 0 \text{ if } CDW < CDW_{dev,a} \\ EN_{wk} \text{ if } CDW >= CDW_{dev,a} \end{cases} \cdot S_{h,a}$

11b. $IHA_{wk+1} = \begin{cases} 0 \text{ if } CDW < CDW_{dev,a} \\ IEA_{wk} \text{ if } CDW >= CDW_{dev,a} \end{cases} \cdot S_{h,a}$

12a. $QA_{wk+1} =$

$$(QA_{wk} - \sum_{x=1}^{3}(QA_{wk} \cdot (a_x \cdot H_x)^b) \cdot N(\mu_{a,a}, \sigma_{a,a})) \cdot (N(\mu_{t,a}, \sigma_{t,a}) \cdot N(\mu_{p,a}, \sigma_{p,a}))$$

12b. $IQA_{wk+1} =$

$$(IQA_{wk} - \sum_{x=1}^{3}(IQA_{wk} \cdot (a_x \cdot H_x)^b) \cdot N(\mu_{a,a}, \sigma_{a,a})) \cdot (N(\mu_{t,a}, \sigma_{t,a}) \cdot N(\mu_{p,a}, \sigma_{p,a}))$$

13a. $OA_{wk+1} = \sum_{x=1}^{3}(QA_{wk} \cdot (a_x \cdot H_x)^b) \cdot N(\mu_{a,a}, \sigma_{a,a}) \cdot (N(\mu_{t,a}, \sigma_{t,a}) \cdot N(\mu_{p,a}, \sigma_{p,a}))$

13b. $IOA_{wk+1} = \sum_{x=1}^{3}(IQA_{wk} \cdot (a_x \cdot H_x)^b) \cdot N(\mu_{a,a}, \sigma_{a,a}) \cdot (N(\mu_{t,a}, \sigma_{t,a}) \cdot N(\mu_{p,a}, \sigma_{p,a}))$

14a. $EA_{wk+1} =$

$$\left(EA_{wk} - \begin{cases} 0 \text{ if } CDW < CDW_{dev,a} \\ EN_{wk} \text{ if } CDW >= CDW_{dev,a} \end{cases}\right) \cdot N(\mu_{t,a}, \sigma_{t,a}) \cdot N(\mu_{p,a}, \sigma_{p,a}) +$$

$$\sum_{x=1}^{3} \left(OA_{wk} \cdot \begin{cases} S_{x,a} \text{ if } EL_{a,wk} > EI_{max} \\ \frac{S_{m,a}-S_{x,a}}{EI_{max}-EI_{min}} \text{ if } EI_{min} < EL_{a,wk} < EI_{max} \\ S_{m,a} \text{ if } EL_{a,wk} < EI_{min} \end{cases} \cdot C_x(1 - \frac{IH_x}{IH_x+H_x}) \right)$$

14a. $IEA_{wk+1} = \left(IEA_{wk} - \begin{Bmatrix} 0 \text{ if } CDW < CDW_{dev,a} \\ IEA_{wk} \text{ if } CDW >= CDW_{dev,a} \end{Bmatrix} \right) \cdot N(\mu_{t,a}, \sigma_{t,a}) \cdot$

$N(\mu_{p,a}, \sigma_{p,a}) + \sum_{x=1}^{3} \left(ION_{wk} \cdot \begin{Bmatrix} S_{x,a} \text{ if } EL_{a,wk} > EI_{max} \\ \frac{S_{m,a} - S_{x,a}}{EI_{max} - EI_{min}} \text{ if } EI_{min} < EL_{a,wk} < EI_{max} \\ S_{m,a} \text{ if } EL_{a,wk} < EI_{min} \end{Bmatrix} \right) +$

$\sum_{x=1}^{3} \left(OA_{wk} \cdot \begin{Bmatrix} S_{x,a} \text{ if } EL_{a,wk} > EI_{max} \\ \frac{S_{m,a} - S_{x,a}}{EI_{max} - EI_{min}} \text{ if } EI_{min} < EL_{a,wk} < EI_{max} \\ S_{m,a}, EL_{a,wk} < EI_{min} \end{Bmatrix} \cdot C_x\left(\frac{IH_x}{IH_x + H_x}\right) \right)$

15a. $EI_{l,wk} = sum_{k=wk}^{wk-9}(0.44^{(k-1)} \cdot sum_{x=1}^{3} \frac{EI_{scl} \cdot (OL_k + IOL_k)}{H_x})$

16a. $SM_{wk+1} =$
$\begin{cases} SM_{wk} \cdot S_x + (SM_{wk} + IM_{wk}) \cdot B_x, wk \ \% \ 52 \neq 0 \\ \begin{cases} SM_{wk} + (SM_{wk} - N(\mu_m, \sigma_m)), SM_{wk} - N(\mu_m, \sigma_m) > 0 \\ SM_{wk} + (SM_{wk} - N(\mu_m, \sigma_m)) \cdot \frac{SM_{wk}}{SM_{wk} + IM_{wk}}, SM_{wk} - N(\mu_m, \sigma_m) < 0 \end{cases} \end{cases}$

16b. $IM_{wk+1} =$
$\begin{cases} IM_{wk} \cdot S_x + T(ITH) \cdot SM_{wk}, wk \ \% \ 52 \neq 0 \\ \begin{cases} IM_{wk}, SM_{wk} - N(\mu_m, \sigma_m) > 0 \\ SM_{wk} + (SM_{wk} - N(\mu_m, \sigma_m)) \cdot \frac{IM_{wk}}{SM_{wk} + IM_{wk}}, SM_{wk} - N(\mu_m, \sigma_m) < 0 \end{cases} \end{cases}$

16c. $SMM_{wk+1} = SMM_{wk} \cdot S_x + (SMM_{wk} + IMM_{wk}) \cdot B_x$

16d. $IMM_{wk+1} = IMM_{wk} \cdot S_x + T(ITH_x) \cdot SMM_{wk}$

16e. $SD_{wk+1} = SD_{wk} \cdot S_x + (SD_{wk} + ID_{wk}) \cdot B_x$

16f. $ID_{wk+1} = ID_{wk} \cdot S_x + T(ITH_x) \cdot SD_{wk}$

17. $ITH_x = TIF \cdot \frac{\sum(IO(L,N,A))}{H_{x,wk} + IH_{x,wk}}$

Note: T is the effective transmission rate, defined in "Infection".
