# Supplementary material for: How do host population dynamics impact Lyme disease risk dynamics in theoretical models?
Source: PLoS One. 2024 May 9;19(5):e0302874. doi: 10.1371/journal.pone.0302874 (PMC11081252; doi:10.1371/journal.pone.0302874)
Supplement: Attachment — Submitted filename: response_to_reviewers.pdf [file pone.0302874.s009.pdf]

Thank you to the editorial board at PLOS ONE and to our reviewer for taking the time to review and comment on this manuscript. We have addressed the points raised by the editor and reviewer. Below are our responses to the raised points.

Editorial points:

1. We have ensured that our mansucript meet's PLOS ONE's style requirements, and made adjustments to fit these requirements as needed.

2. We have removed funding information from the acknowledgements.

3. We acknowledge our data availability statement, and will be maintaining it. We will provide relevant information for publicly accessing data prior to publication.

4. Note an update to our financial disclosure in the cover letter.

Reviewer Points:

1. **"Conceptual structure would help place the results in a ecological context."**
We have addressed this point by taking the suggested example, and reviewing the manuscript to improve the ecological context of our results. Adjustments have been made to the structure and language of the manuscript to further this goal.

2. **"In general, the manuscript would be greatly improved by developing clear research questions and hypotheses"; and 3. The manuscript needs more biological context**
We have addressed these points by restructuring the introduction to the manuscript, adding clearer language, and improving the biological context of our research goals throughout the manuscript. We believe that the manuscript now better reflects the significance of our questions and how we aimed to investigate them.

4. **Formatting.**
We have verified that our formatting follows PLOS ONE's submission standards, apologies for any difficulties in legibility.

5. **Missing detail in the manuscript/lack of polish.**
The errors indicated by our reviewer have been corrected, and we have made further edits to the manuscript to add polish and important details.

6. **"The finding that tick density tracks host density is interesting....Then later on lines 222, the authors seem to contradict their finding that density of hosts is a poor predictor of disease risk based on other studies 17, 20. How do the authors reconcile their results with these other results?"**
We have clarified this section in the manuscript, removing some ambiguous language that could have led to this conclusion. To clarify briefly, the density of hosts has been found to be a poor predictor of infection prevalence, one measure of disease risk. However, the density of hosts is a good predictor of the density of ticks and infected ticks. The previous draft did not make clear which measure of disease risk was being referred to, and this has been corrected. There are lags in the model, but we have focused on a broader look at disease risk measures predicted by the model with or without host dynamics.

7. **Suggested edits and typos.**
We adpoted corrections for the typos and clarifications suggested by the reviewer, and edited the manuscript further to increase clarity and remove typos.

In addition to these edits, we have reviewed the manuscript and made general edits to improve the clarity of the manuscript, and address concerns on the polish and biological context of the manuscript and research goals. We are grateful for the opportunity to submit this manuscript for further review.
